# Influence of Integrated Management Strategies on Soybean Sudden Death Syndrome (SDS) Root Infection, Foliar Symptoms, Yield and Net Returns

**DOI:** 10.3390/pathogens12070913

**Published:** 2023-07-06

**Authors:** Mariama T. Brown, Daren S. Mueller, Yuba R. Kandel, Darcy E. P. Telenko

**Affiliations:** 1 Department of Botany and Plant Pathology, Purdue University, West Lafayette, IN 47907, USA; 2 Department of Plant Pathology, Entomology, and Microbiology, Iowa State University, Ames, IA 50011, USA; 3 5Metis Inc., Research Triangle Park, Durham, NC 27709, USA

**Keywords:** seed treatment, cultivar selection, seeding rate, fluopyram, pydiflumetofen

## Abstract

Three soybean field trials were conducted in Indiana to evaluate the integration of seed treatment, cultivar selection, and seeding rate on sudden death syndrome (SDS) root rot, pathogen load in the root, foliar symptoms, yield, and net return. Two soybean cultivars, one moderately resistant and one susceptible to SDS, were planted at three seeding rates (272,277 seeds/ha, 346,535 seeds/ha, and 420,792 seeds/ha). Fluopyram and pydiflumetofen seed treatments were applied to both cultivars, and the cultivars were then compared with a control. Low foliar SDS disease pressure was observed in our study. Seed treatment with either fluopyram or pydiflumetofen and the use of a moderately resistant cultivar decreased *Fusarium virguliforme* DNA concentration in the root relative to the control and the use of a susceptible cultivar. Fluopyram significantly reduced visual root rot severity by 8.8% and increased yield by 105 kg/ha relative to the control but was not different from pydiflumetofen. However, pydiflumetofen performed the same as the control with respect to root rot severity and yield. Findings from this study support the use of a seed treatment to protect roots from infection and the use of a moderately resistant cultivar planted at a seeding rate of 346,535 seeds/ha to protect yield and maximize net returns when a field has low foliar SDS pressure.

## 1. Introduction

Sudden death syndrome (SDS) is one of the top yield-reducing diseases of soybean [1,2,3]. This disease can cause as much as 100% yield loss, depending on environmental conditions, the age of plants at the time of infection, and the severity of the disease [4,5,6]. *Fusarium virguliforme*, a soilborne fungus, is the main causal agent of SDS in North America [7]. SDS was first observed in 1971 in Arkansas by H. J. Walters [8] and has now become a widespread disease that is found in all main soybean production zones of the U.S [7]. SDS is more prominent under favorable environmental conditions such as a cool and moist spring [9]. Early in the growing season, *F. virguliforme* infects soybean roots, causing root and crown rot; however, the development of foliar symptoms usually begins when the plant enters the beginning pod (R3) and full pod (R4) reproductive stages [10,11,12]. Initially, foliar symptoms appear as interveinal chlorosis, but if the cultivar is susceptible to SDS and soil moisture is favorable, then these symptoms may progress to interveinal necrosis and cause premature defoliation [13,14]. Severe SDS infection can also lead to pod and seed abortion and whole-plant death [4,6]. 

There is no single method that growers can implement to completely manage SDS disease, and so effective management of SDS is achieved through an integrated approach that combines methods that effectively complement each other [4,15]. The adoption of resistant cultivars is a common strategy [16]. Currently, there are no cultivars that are completely resistant to SDS, so the use of a partially resistant cultivar is encouraged [17,18,19,20,21]. However, partial resistance can potentially be overcome by high inoculum densities [19,22,23]. The implementation of cultural practices, such as strategic or occasional tillage, crop rotation, water drainage, and adjustment of planting date, can reduce SDS disease inoculum [11,24,25,26]. Crop rotation has been used to manage many soilborne pathogens, but the use of this strategy has shown inconsistent effects on SDS severity and *F. virguliforme* soil populations [6,24,27]. *F. virguliforme* has a broad host range, which means that crops other than soybeans, such as corn, wheat, and alfalfa, can retain or accumulate inoculum in the soil; thus, crop rotation is not recommended as a management method for SDS [28]. A delay in planting can also reduce the severity of SDS disease, but this may cause a negative impact on yield [6]. In addition, fungicide seed treatment can inhibit early *F. virguliforme* infection, which can prevent SDS disease development later in the season [29]. These SDS management strategies have had mixed results [14,15,24,26,29,30,31,32,33,34,35,36], and improvement in their efficiency is needed because of a lack of complete resistance in commonly grown cultivars, an increased risk for fungicide resistance, and changes in environmental conditions.

Fluopyram (ILEVO, BASF, Research Triangle Park, NC, USA) and pydiflumetofen (Saltro, Syngenta, Research Triangle Park, NC, USA) are registered as fungicide seed treatments for SDS management. Both seed treatments belong to the succinate dehydrogenase inhibitors (SDHI, Fungicide Resistance Action Committee (FRAC) 7 group) [37]. Fluopyram has been available since 2014 and is effective in reducing SDS root rot and foliar symptoms [14,15,38]. However, pydiflumetofen was only recently introduced in 2020 and there is limited published data on its effect on SDS. In addition to the use of fungicide seed treatments, another strategy that can be applied to manage the disease is the use of optimum seeding rates that can reduce the severity of the disease [39,40,41,42]. However, the combination of different seeding rates and seed treatment on *F. virguliforme* infection of the root is not well known. The objective of this study was to evaluate the integration of cultivar selection, seed treatments, and seeding rates on *F. virguliforme* root rot, pathogen load in the root, SDS foliar symptoms, and yield in Indiana. Our goal was to determine the economic impact of these integrated management strategies in order to provide growers in Indiana with a cost-effective management program for SDS.

## 2. Materials and Methods

### 2.1. Field Experiments and Treatments 

Field experiments were established in Wanatah, IN, at the Pinney Purdue Agricultural Center (PPAC) in 2019 and 2020, and in West Lafayette, IN, at the Agronomy Center for Research and Education (ACRE) in 2020. All three field sites had a history of SDS. The experimental design was a randomized split-plot arrangement with the main plot seeding rate and the subplot of factorial cultivars (moderately resistant or susceptible to SDS) by fungicide seed treatments with four replications. Field trial information on soybean cultivar, planting date, previous crop, *F. virguliforme* inoculation, irrigation, presence of SDS foliar symptoms, and harvest date is found in Table 1. Plots were 3.0 m wide (10 ft) and 9.1 m l30 ft), consisting of four rows, with the two center rows used for SDS foliar symptom evaluation and yield and the outer two rows used for destructive root sampling. An SDS moderately resistant (rating 7) and an SDS susceptible (rating 2) soybean cultivar were planted in 76.2 cm (30 in) row spacing at seeding rates of 272,277 seeds/ha, 346,535 seeds/ha, and 420,792 seeds/ha. Soybean cultivars P24A99X and P25A27X were used as the moderately resistant cultivars and P22T24X and P24T76E were the susceptible cultivars and grouped according to their SDS rating for analysis. Seed treatments of fluopyram (ILEVO, BASF, Research Triangle Park, NC, USA) applied at 0.15 mg ai/seed and pydiflumetofen (Saltro, Syngenta, Research Triangle Park, NC, USA) applied at 0.075 mg ai/seed were applied to both cultivars and were compared with the control for SDS. A base seed treatment of metalaxyl (Allegiance Fl at 4.0 g ai/100 kg seed, Bayer Crop Science St. Louis, MO, USA), pyraclostrobin (Stamina at 7.5 g ai/100 kg seed, BASF), fluxapyroxad (Systiva XS Xemium Brand at 5.0 g ai/100 kg seed, BASF), and clothianidin (Poncho 600 at 0.11 mg ai/seed) was added to all treatments in 2020 to minimize nontarget pathogen and insect pest pressure. These active ingredients were previously known to have no effect on SDS [14,15,23,29]. Plots were artificially infested with *F. virguliforme* inoculum following the procedure described by de Farias Neto et al. [43]. Inoculum was prepared from local Indiana isolates (INMOG4) of *F. virguliforme*, which was used to colonize sterilized sorghum. Infested sorghum at 4.1 g/m was placed in-furrow at planting. Irrigation was applied at the Wanatah location in both years to encourage SDS development, and the field was overhead irrigated weekly at 25 mm (1 in.) unless weekly rainfall was 1 in. or higher (Table 1).

### 2.2. Disease and Yield Data Collection

Root rot severity, foliar SDS incidence and severity, and yield data were recorded from each research plot. The plant population was recorded to validate the different seeding rates used, but it was not evaluated as a response variable. Root rot rating was assessed at the full pod (R4) growth stage. Ten soybean roots were arbitrarily selected and dug from the outside rows (non-yield rows) of each plot, washed, and root rot severity was visually assessed as a percentage (0–100%) of dark discoloration on roots [44]. SDS was rated for foliar disease incidence (DI) and disease severity (DS) in the middle two rows of each plot at full seed to beginning maturity (R6 to R7) growth stages. DI refers to the percentage of plants with SDS foliar disease symptoms, and disease severity (DS) was rated using a 1–9 scale where 1 refers to low foliar disease pressure and 9 refers to the premature death of the plant [45]. The foliar SDS disease index (FDX) was then calculated using the equation FDX = DI × DS/9. The two center rows of each plot were harvested with a Kincaid 8XP plot combined, and yields were calculated and adjusted to 13% moisture prior to analysis. 

### 2.3. Quantification of Fusarium virguliforme DNA in Roots 

After the root rot rating, the roots were air-dried in a drying room at 32 °C for one week. The ten dried roots that were collected from each plot were ground to a fine powder using an Eberbach cutting mill (E3703) with a 0.50 mm sieve size (Eberbach Corporation, Belleville, MI, USA). To avoid cross-contamination between plot samples, the grinder was sprayed with 70% ethanol and wiped dry with a paper towel. DNA was extracted from 100 mg of ground root tissue from each plot sample using a DNeasy Plant Mini Kit (Qiagen, Germantown, MD, USA) according to the manufacturer’s recommendations. DNA quantity and quality were determined using a Nanodrop 2000 spectrophotometer (Thermo Fisher Scientific, Wilmington, DE, USA) and adjusted to 10 ng/μL. Amplifications were performed on CFX96 Touch™ Real-Time PCR Detection System (Bio-Rad Laboratories, Inc., Hercules, CA, USA). The PCR mix and thermal conditions were conducted as described by Wang et al. [46]. Real-time PCR was conducted in a 96-well plate with three technical replications of each DNA sample from each plot. Three non-template water controls were included in each plate along with eighteen samples of *F. virguliforme* DNA serial dilutions (10-fold from 5 ng to 50 fg) for the standard curve. The standard curve was used to determine *F. virguliforme* DNA concentration in each unknown sample. Quantities of *F. virguliforme* DNA were calculated in picograms per 20 ng total DNA.

### 2.4. Partial Profit Analysis

To determine the profitability of the integrated management strategies for SDS, a partial profit analysis was carried out using the equation = (yield × grain sale price) − (seed cost + seed treatment cost). A soybean grain sale price of $0.37/kg ($10.15/bu) was used, which was the average price received for soybean in the U.S. from 2019 to 2021 according to the USDA National Agricultural Statistics Service [47,48]. The grain sale price was multiplied by yield to determine a predicted income. The cost of seed was approximated to be $64 unit-1 (140,000 seeds) according to regional seed costs in 2021 obtained from a local Indiana seed company. Based on this approximation, a final seed cost was determined for all seeding rates examined in this study. According to a local crop protectant dealer, the cost of applying fluopyram seed treatment was approximated at $13 unit-1 (140,000 seeds) and the cost of pydiflumetofen was approximated at $14 unit-1 (140,000 seeds). All other associated costs were assumed to be equal across all treatments and were excluded from this analysis. 

### 2.5. Environmental Data

Environmental data for each location were downloaded from Purdue Automated Agricultural Weather Stations (PAAWS) through the Midwestern Regional Climate Center’s online weather database (mrcc.purdue.edu). Data were collected from weather stations near each research site in Wanatah and West Lafayette, IN, and include dates that considered the growing season for each year (May 1 to October 31). The Wanatah weather station was approximately 1.2 km from the study field while the West Lafayette weather station was approximately 1.3 km from the study field. In addition, data normals for the respective months were downloaded to consider the overall deviation from the norm for each year. Weather data norms were based on the calculated norms between 1991 and 2020, which encompassed the most recent 30-year normal. The specific environmental data considered from each location were temperature minimum, maximum, and mean (°C), and rainfall (cm).

### 2.6. Data Analysis 

All data were analyzed in SAS (version 9.4; SAS Institute, Inc., Cary, NC, USA). A generalized linear mixed model analysis of variance was performed using PROC GLIMMIX. Cultivars, seed treatments, seeding rates, and their interactions were treated as fixed factors, and location and replication were treated as random factors. For root rot severity, grain yield, and net returns, a normal distribution was used along with Kenward-Rogers degrees of freedom. Least-squares means (lsmeans) of the treatments were computed and compared (α = 0.05). All pairwise differences among lsmeans were compared only if the F test was significant (*p* ≤ 0.05) [49]. Foliar Disease Index (FDX) and *F. virguliforme* DNA concentration values were log-transformed prior to analysis to normalize data; back-transformed means are presented.

## 3. Results and Discussion

### 3.1. Environment

The environment varied between the two locations and years during the duration of the research (Table 2). In Wanatah, the deviation in temperature minimum and the 30-year norm was lower for seven of the trial months. Conversely, the deviation in temperature maximum and the 30-year norm was greater for five of the months, with greater than 30-year average temperatures occurring in five months in Wanatah (Table 2). In West Lafayette in 2020, deviations in minimum and maximum temperatures were lower in four of the six months, and the average temperature was greater than the 30-year norm for two of the six months (Table 2). An increase in rainfall compared with the 30-year norms was recorded in Wanatah during May, June, and September 2019 and May 2020, and the increase ranged from 0.6% to 46.5%. However, rainfall decreased in Wanatah during July and August 2019 and from June through October 2020. Rainfall was below the 30-year norm for the entire season in 2020 in West Lafayette and corresponded to the drought conditions that were observed that year (Table 2).

### 3.2. Effect of Cultivar Selection, Seed Treatment, and Seeding Rate on Root Rot and F. virguliforme Pathogen Load in the Root

There were no significant interactions among cultivars, seed treatments, and seeding rates with respect to root rot severity (Table 3), therefore main effects were examined. Cultivar selection significantly influenced root rot symptom development (*p* = 0.0021) (Table 3). The use of a susceptible cultivar significantly reduced root rot severity by 7.8% when compared with the use of a resistant cultivar (Table 4). This result is similar to what has been reported previously, in which the moderately resistant cultivar had more root rot than the moderately susceptible cultivar in one research location [15]. A recent study also found that the moderately resistant cultivar had greater root rot than the susceptible cultivar in one study year [50]. These results may be explained by the quantitative trait loci (QTLs) that govern SDS resistance, which adds to root rot or foliar resistance singly [51,52]. Foliar symptoms are the main reason for yield loss in SDS-susceptible soybean lines [53] and so a phenotypic rating that is used for SDS resistance relies mostly on the basis of foliar symptoms rather than root rot symptoms [54]. 

Root rot severity was also significantly influenced by seed treatments (*p* = 0.0072) (Table 3). The fluopyram seed treatment significantly reduced root rot severity by 8.8% when compared with the control, but it was not significantly different from the pydiflumetofen seed treatment. However, the pydiflumetofen seed treatment was not significantly different from the control with respect to root rot severity (Table 4). Pydiflumetofen seed treatment was only recently marketed for SDS management, so there is limited published data on its efficacy for root rot reduction. Previous studies have reported that fluopyram seed treatment was effective for reducing root rot severity [14,15,23,38], while a recent study has reported that both fluopyram and pydiflumetofen were most effective for managing SDS [50]. 

The concentration of *F. virguliforme* DNA in soybean roots was used as a measure of SDS pathogen DNA concentration. There were no significant interactions among cultivars, seed treatments, and seeding rates with respect to the concentration of *F. virguliforme* DNA in soybean roots (Table 3), therefore main effects were examined. *F. virguliforme* DNA concentration in the root was significantly influenced by cultivar (*p* = 0.0092) (Table 3). Our results suggest that using a moderately resistant cultivar could significantly decrease *F. virguliforme* DNA concentration in the root by 37.5% relative to the use of a susceptible cultivar (Table 4). Similarly, Wang et al. [55] found that at 35 days after planting, there was a significantly higher *F. virguliforme* infection coefficient for susceptible cultivars relative to the moderately resistant cultivars. No cultivar is completely resistant to SDS disease. Nevertheless, the use of a moderately resistant cultivar can be an effective tactic and should be the primary means for managing SDS disease [56]. 

*F. virguliforme* DNA concentration in the root was also significantly reduced by the use of seed treatments (*p* = 0.0001) (Table 3). Fluopyram and pydiflumetofen seed treatments significantly reduced *F. virguliforme* DNA concentration in the root by 75% and 64% relative to the control, respectively (Table 4). In a previous study, the fluopyram seed treatment was also reported to be effective in reducing *F. virguliforme* root infection [38]. Seed treatment protects the seeds from early infection, which then limits the development of SDS symptoms later in the growing season [29]. Therefore, seed treatments should be included as part of an effective management strategy for SDS disease.

Different seeding rates can impact disease development, yield, and net return of a crop [40,41,42,57,58,59,60,61]. However, there is limited published data on the effects of different seeding rates on SDS development. From our trials in Indiana, seeding rates did not have a significant effect on SDS root rot severity (*p* = 0.0563) or *F. virguliforme* pathogen load in the root (*p* = 0.8363) (Table 4). Similarly, Kandel et al. [62] found that the seeding rate did not affect stem diseases of soybeans. In contrast, a greater incidence of Sclerotinia stem rot, tomato spotted wilt, and peanut stem rot were observed under higher seeding rates [41,42,63].

### 3.3. Effect of Cultivar Selection, Seed Treatment, and Seeding Rate on Foliar Symptoms

In 2019 and 2020, SDS foliar symptoms were observed at the Wanatah location, but at low levels <1.0 SDS foliar disease index (FDX). No SDS foliar symptoms were observed at the West Lafayette location in 2020, and no other foliar or stem disease was noted in any of the experiments. There was no significant main effect of cultivar selection, seeding rate, or seed treatment for SDS FDX. However, there was a significant interaction between cultivar and seeding rate for FDX (*p* = 0.0378) (Table 3). A moderately resistant cultivar planted at 272,277 seeds/ha or 346,535 seeds/ha significantly reduced FDX when compared with a susceptible cultivar planted at 346,535 seeds/ha but did not significantly reduce FDX when compared with a susceptible cultivar planted at 272,277 seeds/ha or 420,792 seeds/ha or a moderately resistant cultivar planted at 420,792 seeds/ha.

### 3.4. Effect of Cultivar Selection, Seed Treatment, and Seeding Rate on Grain Yield and Partial Profit

Grain yield was significantly impacted by seed treatment (*p* = 0.0435) (Table 5). The fluopyram seed treatment produced the most yield with a 105 kg ha-1 significant increase relative to the control but was not significantly different from pydiflumetofen. However, the pydiflumetofen seed treatment was not statistically different from the control for grain yield (Table 5). In addition, there was a significant two-way interaction effect between cultivar and seeding rate for grain yield (*p* = 0.0484) (Table 3). A moderately resistant cultivar planted at a seeding rate of 346,535 seeds/ha significantly increased yield over a susceptible cultivar that was planted at 272,277 or 346,535 seeds/ha. However, it was not statistically different in yield when compared with a susceptible cultivar planted at a seeding rate of 420,792 seeds/ha (Table 5). This result suggests that when planting a susceptible cultivar, a seeding rate of 420,792 seeds/ha should be considered to recover the same amount of grain yield as from a moderately resistant cultivar planted at a seeding rate of 346,535 seeds/ha. 

Net returns were also significantly impacted by the interaction effect of cultivar and seeding rate (*p* = 0.0484) (Table 5). A moderately resistant cultivar planted at 346,535 seeds/ha gave a significant increase in net returns when compared with a susceptible cultivar planted at 272,277 or 346,535 seeds/ha but was not statistically different from a moderately resistant cultivar planted at a seeding rate of 272,277 seeds/ha or a susceptible cultivar planted at 420,792 seeds/ha. Although they produced similar grain yields, a moderately resistant cultivar planted at 346,535 seeds/ha had a significant increase in net returns when compared with a moderately resistant cultivar planted at 420,792 seeds/ha. This response in net returns is likely due to the increase in the input cost of planting more seeds in a field with low foliar SDS disease pressure. In contrast, net returns were not significantly impacted by seed treatment (*p* = 0.1039) (Table 5), even though it affected grain yield. These results suggest that under low foliar SDS disease pressure, yield gain from a seed treatment may not be sufficient to cause an increase in net returns.

## 4. Conclusions

This study evaluated the integration of disease management tactics that included pydiflumetofen and fluopyram seed treatments, moderately resistant and susceptible cultivars, and different seeding rates on SDS development, yield, and net returns. This work demonstrates that using a seed treatment that includes fluopyram or pydiflumetofen is important in protecting soybean roots from *F. virguliforme* infection. In a field with low foliar SDS disease pressure, the fluopyram seed treatment significantly reduced root rot severity and increased grain yield when compared with the control, but it was not significantly different from the pydiflumetofen seed treatment. However, the pydiflumetofen seed treatment performed the same when compared with the control for root rot severity and grain yield. In addition, our findings suggest that in low SDS disease environments, a moderately resistant cultivar planted at a seeding rate of 346,535 seeds/ha should be considered for an increase in grain yield and subsequent increase in net returns. However, if planting a susceptible cultivar under these environments, a seeding rate of 420,792 seeds/ha should be considered to increase grain yield and net returns. There is a continued need to use an integrated management approach for SDS that includes resistance, seed treatment, and optimum seeding rate. Therefore, future research should continue to evaluate new products or methods for effective SDS management using an integrated approach. 

## Figures and Tables

**Table 1:** Field locations and trial details used for integrated disease management experiments of sudden death syndrome (SDS) in soybean in Indiana.

Year	Location ^a^	Cultivars ^b^	Planting Date	Previous Crop	Inocu-lation ^c^	Irrigated ^d^	SDS Foliar Symptoms	Harvest Date
2019								
	Wanatah, IN	P24A99X (MR) P22T24X (S)	6/6	Corn	Yes	Yes	Yes	10/24
2020								
	Wanatah, IN	P25A27X (MR) P24T76E (S)	6/6	Corn	Yes	Yes	Yes	11/02
	West Lafayette, IN	P25A27X (MR) P24T76E (S)	5/13	Soybean	Yes	No	No	10/14

^a^ Trials located in Wanatah, IN, at Pinney Purdue Agricultural Center (PPAC) and in West Lafayette, IN, at Agronomy Center for Research and Education (ACRE). ^b^ MR = SDS moderately resistant cultivar; S = SDS susceptible cultivar. ^c^ Field experiments were inoculated in furrow at planting with Indiana *F. virguliforme* isolates that had been used to colonize sterilized sorghum at 4.1 g/m within the seedbed. ^d^ Overhead irrigation was applied weekly at 25 mm (1 in.) unless weekly rainfall was 25 mm or greater in 2019 and 2020.

**Table 2:** Environmental variables presented as the minimum, maximum, and average temperatures (°C) and precipitation (cm) for each location and year along with the deviation from the 30-year normal for locations where trials were conducted in Indiana during 2019 and 2020.

	Monthly Minimum, Maximum, and Average Temperature (°C) and Rainfall Received (cm). Deviation from the 30-Year Normal in Parentheses ^a,b^							
	Wanatah, Indiana				West Lafayette, IN			
	Tmin	Tmax	Tave	Rainfall	Tmin	Tmax	Tave	Rainfall
2019								
May	9.0 (−0.4)	19.9 (−1.6)	14.4 (−1.1)	18.9 (8.8)	-	-	-	-
June	14.2 (−0.8)	25.4 (−1.4)	19.8 (−1.1)	12.8 (0.8)	-	-	-	-
July	17.9 (1.4)	29.6 (1.2)	23.8 (1.3)	4.8 (−5.8)	-	-	-	-
August	14.6 (−0.6)	27.1 (−0.3)	20.8 (−0.4)	6.7 (−4.5)	-	-	-	-
September	13.6 (2.8)	25.4 (0.9)	19.5 (1.9)	17.5 (9.2)	-	-	-	-
October	4.8 (0.1)	16.4 (−1.0)	10.6 (−0.5)	11.0 (1.4)	-	-	-	-
2020								
May	9.1 (−0.4)	19.1 (−2.4)	14.1 (−1.4)	17.3 (7.1)	9.7 (−0.8)	20.2 (−2.2)	14.9 (−1.4)	8.4 (−3.5)
June	15.9 (0.8)	28.5 (1.7)	22.2 (1.3)	10.7 (−1.3)	16.1 (0.3)	28.4 (1.3)	22.2 (0.8)	6.3 (−6.0)
July	17.8 (1.3)	29.6 (1.2)	23.7 (1.2)	9.4 (−1.2)	18.0 (1.1)	29.6 (1.2)	23.8 (1.1)	10.2 (−0.6)
August	14.9 (−0.3)	27.8 (0.5)	21.4 (0.1)	4.5 (−6.6)	15.2 (−0.4)	27.3 (−0.3)	21.2 (−0.4)	8.7 (−0.6)
September	10.3 (−0.4)	23.9 (−0.6)	17.1 (−0.5)	5.5 (−2.8)	10.9 (−0.3)	24.4 (−0.6)	17.7 (−0.4)	5.6 (−2.3)
October	4.0 (−0.7)	15.9 (−1.5)	10.0 (−1.1)	6.6 (−3.0)	4.2 (−0.1)	17.2 (−0.9)	10.7 (−1.0)	6.1 (−1.6)

^a^ Values are presented as the difference between the average temperature and the total rainfall for each month within each year and compared with the 30-year normal (1991 to 2020) for each location in Indiana. ^b^ Values were downloaded from Purdue Automated Agricultural Weather Stations (PAAWS) as part of the Midwestern Regional Climate Center (mrcc.purdue.edu) for each of the locations, Wanatah and West Lafayette, Indiana, where soybean trials were conducted.

**Table 3:** Results of main effects and interactions for root rot, *Fusarium virguliforme* pathogen load in the root (FPL), foliar symptoms (FDX), yield, and net return for three field trials across Indiana in 2019 and 2020.

	*p* Value				
Effect	Root Rot	FPL ^a^	FDX ^a^	Yield	Net Return
Cultivar	0.0021	0.0092	0.1188	0.0213	0.0213
Seed treatment	0.0072	0.0001	0.9952	0.0435	0.1039
Seeding rate	0.0563	0.8363	0.4682	0.0461	0.7175
Cultivar × seed treatment	0.8000	0.8104	0.2868	0.2075	0.2074
Cultivar × seeding rate	0.6145	0.9727	0.0378	0.0484	0.0484
Seed treatment × seeding rate	0.4573	0.2945	0.4640	0.3668	0.5666
Cultivar × seed treatment × seeding rate	0.6541	0.7545	0.4964	0.2565	0.2565

^a^ FPL = *Fusarium virguliforme* pathogen load in the root (Quantification of *F. virguliforme* DNA in roots), and FDX = Foliar disease index.

**Table 4:** Effect of cultivars, seed treatments, and seeding rates on sudden death syndrome (SDS) root rot severity, *Fusarium virguliforme* pathogen load in the root (FPL), and foliar disease index (FDX) in Indiana ^v^.

Effect	Root Rot (%) ^w^	FPL (pg/20 ng DNA) ^x^	FDX (%) ^y^
**Cultivars**			
Moderately resistant	28.2 a ^z^	1.0 b	0.2
Susceptible	26.0 b	1.6 a	0.3
*p* value	0.0021	0.0092	0.1188
**Seed treatments**			
Control	28.3 a	2.8 a	0.2
Fluopyram	25.8 b	0.7 b	0.2
Pydiflumetofen	27.2 ab	1.0 b	0.2
*p* value	0.0072	0.0001	0.9952
**Seeding rates**			
272,277 seeds/ha	26.0	1.2	0.2
346,535 seeds/ha	27.2	1.2	0.3
420,792 seeds/ha	28.1	1.4	0.3
*p* value	0.0563	0.8363	0.4682

^v^ Seed treatments with fluopyram (ILEVO, BASF, Research Triangle Park, NC, USA) applied at 0.15 mg ai/seed and pydiflumetofen (Saltro, Syngenta, Research Triangle Park, NC, USA) applied at 0.075 mg ai/seed. ^w^ Root rot was visually assessed as a percentage of dark discoloration on roots at the full pod (R4) growth stage. ^x^ Fusarium virguliforme pathogen load in the root was estimated using an F. virguliforme-specific quantitative polymerase chain reaction assay in picograms/nanogram (pg/ng) [46]. ^y^ Sudden death syndrome (SDS) in each plot was rated for disease incidence (DI) and disease severity (DS) at the full seed to beginning maturity (R6–R7) growth stages. Disease incidence was the percentage of plants with disease symptoms (0–100%), and disease severity (DS) was rated using a 1–9 scale where 1 refers to low disease pressure and 9 refers to the premature death of the plant. SDS foliar disease index (FDX) was then calculated using the formula, FDX = (DI × DS)/9 [45]. ^z^ Values are least-squares means. Values with different letters in each column are significantly different based on the least-squares means test (α = 0.05).

**Table 5:** Effect of seed treatment and cultivar by seeding rate on soybean yield and net returns in Indiana.

Effect		Yield (kg/ha) ^w^	Net Return ($/ha) ^x^
**Seed treatment** ^y^			
Control		3879 b ^z^	1277
Fluopyram		3984 a	1284
Pydiflumetofen		3882 ab	1243
*p* value		0.0435	0.1039
**Cultivar resistance type**	**Seeding rates**		
Moderately Resistant	272,277 seeds/ha	3864 bcd	1288 ab
Moderately Resistant	346,535 seeds/ha	4044 a	1316 a
Moderately Resistant	420,792 seeds/ha	3975 abc	1252 b
Susceptible	272,277 seeds/ha	3774 d	1255 b
Susceptible	346,535 seeds/ha	3829 cd	1236 b
Susceptible	420,792 seeds/ha	4002 ab	1262 ab
*p* value		0.0484	0.0484

^w^ Yields were adjusted to 13% moisture at harvest prior to analysis. ^x^ A partial profit analysis was used to calculate the expected net return using the equation = (Yield × Grain Sale Price) − (Seed cost + seed treatment cost). A soybean grain sale price of $0.37 kg^−1^ ($10.15 bu^−1^) was used. ^y^ Seed treatments of fluopyram (ILEVO, BASF, Research Triangle Park, NC, USA) applied at 0.15 mg ai/seed and pydiflumetofen (Saltro, Syngenta, Research Triangle Park, NC, USA) applied at 0.075 mg ai/seed. ^z^ Values are least-squares means. Values with different letters are significantly different based on the least-squares means test (α = 0.05).

## Data Availability

At the time of submission, the authors have not yet made these data publicly available. Data will be made publicly available at https://purr.purdue.edu/publications/4281/1 (accessed on 25 June 2023) upon full acceptance of the manuscript.
